# Transmission of psoriasis by allogeneic bone marrow transplantation and blood transfusion

**DOI:** 10.1038/bcj.2015.15

**Published:** 2015-03-13

**Authors:** X Li, J Li, L Wang, X Niu, R Hou, R Liu, Z Hao, C Wang, G Yin, K Zhang

**Affiliations:** 1 Institute of Dermatology, Taiyuan City Centre Hospital, Taiyuan, China; 2 Department of Dermatology, Shanxi Academy of Medical Sciences, Taiyuan, China; 3 Department of Dermatology, General Hospital of TISCO, Taiyuan, China; 4 Laboratory Animal Center, Shanxi Medical University, Taiyuan, China

Psoriasis is an immune-mediated dermatological disease, with T-cells have an important role in its pathogenesis. Activated CD4^+^ and CD8^+^ lymphocytes infiltrate the dermis and epidermis, resulting in hyperkeratosis, parakeratosis, epidermal acanthosis, elongation of the rete ridges and vascular dilatation. Before allogeneic bone marrow transplantation (BMT), the immune system of the host is effectively eliminated by a preconditioning regimen, and any immune response after BMT is typically of donor origin.^1, 2^ In the past 25 years, >30 patients with psoriasis who underwent BMT and subsequently achieved long-term remission of psoriasis have been reported in the literature.^3, 4^ Interestingly, patients who developed psoriasis following allogeneic BMT have also been reported, with three cases of acquired psoriasis after BMT from donors with psoriasis.^5, 6, 7^ We report two additional cases of patients who developed psoriasis after blood transfusion or allogeneic BMT.

Case one: A 29-year-old woman was admitted for inpatient treatment on 20 August 2006. Three months before, the patient had received whole blood, due to postnatal hemorrhea, from a type- and Rh-matched donor who had a 10-year history of psoriasis vulgaris. The patient's erythra was diffusely distributed, of unequal size, scaled and positive for Auspitz's sign (Figure 1a). A biopsy of the lesion revealed a thick epidermis with the typical pattern and Munro–Sabouraud microabscesses (Figure 1b). On the basis of the clinical and biopsy data, the patient was diagnosed with psoriasis vulgaris. After 5 weeks of combined treatment with penicillin, sodium thiosulfate, vitamin B12 and folic acid, the lesions resolved. The patient has been psoriasis-free through August 2014.

Case two: A 35-year-old man with ETO (runt-related transcription factor 1; translocated to, 1 (cyclin D-related)) fusion gene-positive (t(8;21)(q22;q22)) acute myelogenous leukemia (AML) visited the dermatology outpatient clinic on 15 May 2014, complaining of a skin lesion. In October 2010, he had been diagnosed with AML-M2/ETO. Neither the patient nor his family suffered from psoriasis, and he was negative for HLA-B27. After treatment with hydroxyurea for 7 months, followed by conditioning therapy with busulfan and cyclophosphamide, BMT was performed from an HLA-matched donor, who had suffered from psoriasis for >10 years. The patient was treated with Bu/CY radiation before BMT (1238 ml of bone marrow containing CD34^+^ cells at 3.01 × 10^8^/kg), and cyclosporine A was prophylactically administered for graft-versus-host disease (GVHD). Three months after BMT, acute grade 3 GVHD occurred, involving the skin and liver, and therefore treatment with prednisone (2 mg/kg) was initiated. Immunosuppression was stopped 1 year after the BMT, and the patient subsequently developed lesions on his chest, back and four limbs. The papules were red, covered with multilayer sliver scales and positive for Auspitz's sign (Figure 1c). A biopsy of the lesion revealed a pattern of hyperkeratosis, parakeratosis, acanthosis, regular extension of the trochanterellus, Munro–Sabouraud's microabscesses and inflammatory cell infiltration into the dermal papilla (Figure 1d). On the basis of the clinical and histopathological data, the patient was diagnosed with psoriasis vulgaris. He was subsequently treated with Compound Indigo Naturalis Capsule (Tianning, Xi'an, China) and Compound Glycyrrhizin Capsules (Kawin, Beijing, China), and the lesions remitted after 1 month of treatment and vanished after 3 months of treatment. However, 6 months later, the lesions developed again, indicating a relapse of the psoriasis. This clinical response indicates that treatment with Compound Indigo Naturalis Capsule and Compound Glycyrrhizin Capsules results in a notable effect.

These two cases highlight the fact that psoriasis can be transferred via BMT and blood transfusions. Given that the pathogenesis of psoriasis relies on constituents of the peripheral blood, it has been suggested that T-cells may be the source of psoriasis after blood transfusions, indicating that T-cells somehow transmit the morbidity factor(s) for psoriasis. However, peripheral T-cells have a relatively short lifespan, which indicates that the morbidity factor(s) for psoriasis is also short-lived, resulting in the psoriasis resolving after blood transfusion.

Combined with previous reports, these cases indicate that BMT can result in both remission and transmission of psoriasis. In contrast to BMT-induced remission, the BMT-related acquisition of psoriasis further supports the existence of principal psoriatic morbidity factor(s). Before BMT, the recipient's immunity is effectively eliminated by a preconditioning regimen and therefore it was unclear whether the BMT-induced remission was the result of immune destruction or the transfer of the donor's immune cells. However, the phenomenon of BMT-related acquisition of psoriasis suggests that psoriasis is caused by the transfer of the donor's immune cells. Psoriasis is known to enter remission after allogeneic, but not autologous, hematopoietic stem-cell transplantation,^4^ indicating that hematopoietic stem cells carry the principal morbidity factor(s) of psoriasis. Our previous studies^8, 9, 10, 11^ have reported the abnormal activity of hematopoietic stem cells and the T-cells that have differentiated from the hematopoietic stem cells. In conclusion, these cases indicate that hematopoietic stem cells and T-cells participate in the pathogenesis of psoriasis.

The patients provided informed consent for this publication, and the manuscript was approved by the appropriate ethics review boards.

## Figures and Tables

**Figure 1:**
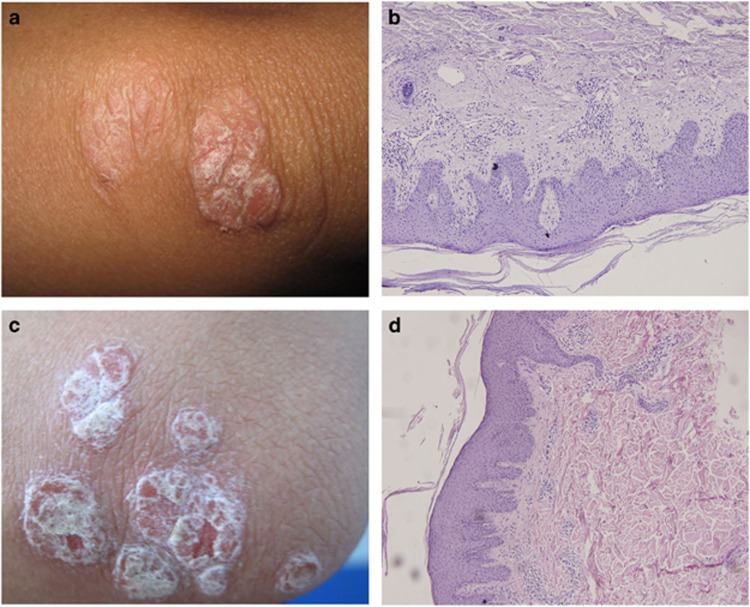
Clinical and histopathological pictures of the patient's lesion after blood transfusion or allogeneic BMT. (**a**) Clinical picture of the patient's lesion after blood transfusion. (**b**) Histopathological picture of the patient's lesion after blood transfusion (HE stained, × 200). (**c**) Clinical picture of the patient's lesion after allogeneic BMT. (**d**) Histopathological picture of the patient's lesion after allogeneic (HE stained, × 200). HE, hematoxylin and eosin.
